# Osmosis-Driven Water Transport through a Nanochannel: A Molecular Dynamics Simulation Study

**DOI:** 10.3390/ijms21218030

**Published:** 2020-10-28

**Authors:** Changsun Eun

**Affiliations:** Department of Chemistry, Hankuk University of Foreign Studies, Yongin 17035, Korea; ceun@hufs.ac.kr

**Keywords:** molecular dynamics simulation, osmosis, water transport, nanochannel, carbon nanotube, graphene, osmolyte, compartment

## Abstract

In this work, we study a chemical method to transfer water molecules from a nanoscale compartment to another initially empty compartment through a nanochannel. Without any external force, water molecules do not spontaneously move to the empty compartment because of the energy barrier for breaking water hydrogen bonds in the transport process and the attraction between water molecules and the compartment walls. To overcome the energy barrier, we put osmolytes into the empty compartment, and to remove the attraction, we weaken the compartment-water interaction. This allows water molecules to spontaneously move to the empty compartment. We find that the initiation and time-transient behavior of water transport depend on the properties of the osmolytes specified by their number and the strength of their interaction with water. Interestingly, when osmolytes strongly interact with water molecules, transport immediately starts and continues until all water molecules are transferred to the initially empty compartment. However, when the osmolyte interaction strength is intermediate, transport initiates stochastically, depending on the number of osmolytes. Surprisingly, because of strong water-water interactions, osmosis-driven water transport through a nanochannel is similar to pulling a string at a constant speed. Our study helps us understand what minimal conditions are needed for complete transfer of water molecules to another compartment through a nanochannel, which may be of general concern in many fields involving molecular transfer.

## 1. Introduction

Water molecules are polar molecules that strongly interact with each other, and they form a hydrogen network, whereas nonpolar molecules do not have such a strong interaction. This difference in the intermolecular interaction strength can be manifested in their physical states and transport behavior. For example, under ambient conditions, water molecules are in the liquid state but their corresponding nonpolar molecules, which are artificial molecules made from water molecules by removing electric charges, show gaseous behavior [1]. Thus, in this case, when we transfer molecules from one compartment to another compartment, water molecules do not spontaneously move to the other compartment without any external forces, while nonpolar molecules can move via diffusion. In particular, for the latter case, we intensively studied the nonequilibrium transport behavior of nonpolar molecules driven by diffusion and osmotic pressure, and the equilibrium states, using molecular dynamics (MD) simulations [1]. However, such detailed research on nonequilibrium water transport has not been performed. Therefore, here, to study the case of water, we use the same system that we employed in our previous work for nonpolar molecules [1] but use water molecules instead of nonpolar molecules. 

Not only is water transport interesting in terms of the need to better understand the fundamental nature of molecular transport for strongly interacting molecules, but also, it is of concern in natural and engineering systems involving water transport. One example of a natural system is that in a cell, water transport occurs to regulate osmolality through aquaporins [2,3,4]. Another example in water engineering is water transport in reverse osmosis to obtain pure water, where water passes through a membrane with carbon nanotubes under high pressure [5]. In these examples, water moves from one location to another. Therefore, understanding the mechanisms by which water moves is essential in controlling water flow in real cases as well as advancing our knowledge. 

Why do water molecules move? To properly address this question, specifically, let us assume we have two nanoscale compartments connected by a nanochannel, in which one compartment is filled with water and the other is empty. How can one transfer all the water molecules from the filled compartment to the empty compartment? In principle, mass transfer, including water transport, occurs when the chemical potential exhibits a spatial difference [6]; thus, the key idea is to create a desirable chemical potential difference. In particular, for the transfer to the empty compartment, one needs to lower the chemical potential in the empty compartment or raise the chemical potential in the filled compartment. For this, one can use a chemical method to create osmotic pressure, such as in aquaporins [4] and carbon nanotube membranes [7], by inserting osmolytes into the empty compartment [1], or use a mechanical method to create a pressure difference, such as in reverse osmosis, by applying external pressure [5,8,9,10]. In this work, we focus on the chemical method and discuss what specific conditions are needed for our system to induce complete water transfer to the empty compartment. We also address the kinetic characteristics during water transport, along with the kinetically stable states. 

This paper is organized as follows. In Section 2, we introduce our model systems to study the water transport and explain the details of MD simulations. In Section 3, we investigate when water transfer occurs by examining the number of osmolytes and their interaction strength with water molecules. We also discuss the kinetically stable and equilibrium states appearing in the transfer due to the osmolytes and the associated kinetic properties such as the waiting time and transition time for the transport, order of the transition rate law, and transport rate. In Section 4, we summarize our findings and the implications of our work. 

## 2. Computational Models and Methods

**Figure 1:**
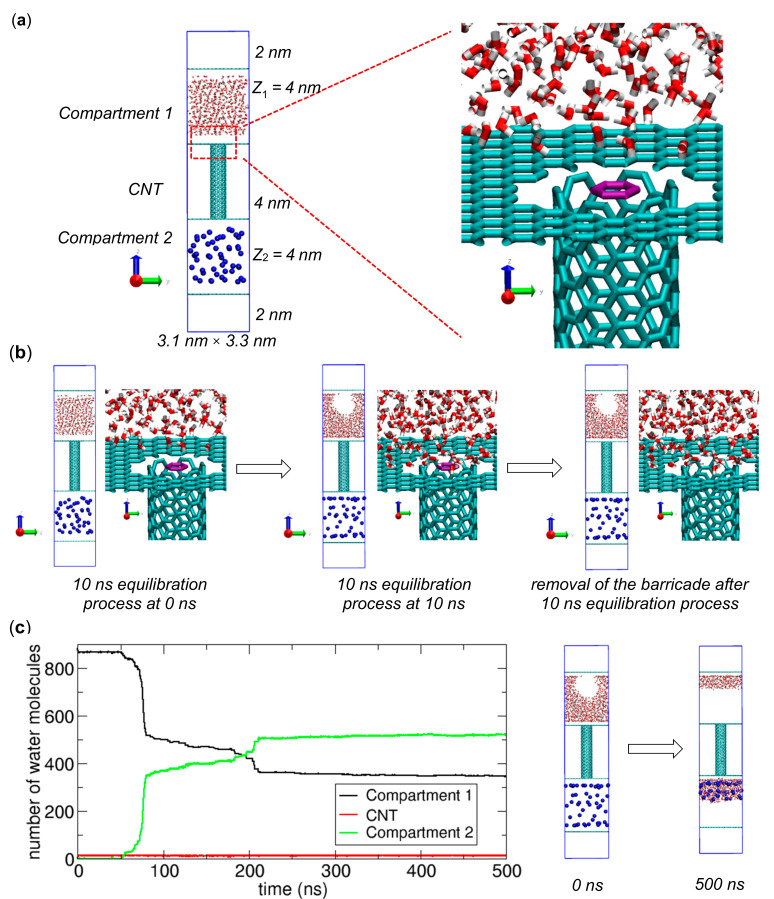
Simulation of water molecule transport from a compartment filled with water molecules (Compartment 1) to another spacious compartment (Compartment 2) through a carbon nanotube (CNT) in the presence of 50 osmolytes with *IS_WO_* = 10. Here, osmolytes are represented by blue spheres. (**a**) Initial configuration of the system, in which all water molecules are in Compartment 1 (left), and a zoomed-in view around the boundary between Compartment 1 and the CNT (right), where a molecular barricade, i.e., a six-carbon ring (violet), is located at a fixed position on the boundary. From the zoomed-in view, a gap appears to exist between the CNT and the graphene with a hole such that water might pass through it, but in reality, the gap is too small for water to pass through it. Note that the blue lines indicate the periodic boundary of the system. (**b**) Configurations at 0 ns (left) and 10 ns (center) from the 10 ns equilibration process in the presence of the barricade (violet), and the configuration (right) in which the six-carbon ring has been removed after the equilibration process. The last configuration is used for the simulation of water transport. (**c**) Numbers of water molecules in Compartment 1 (black), the CNT (red), and Compartment 2 (green) during a transport process (left) along with MD simulation snapshots at 0 and 500 ns (right).

To study the transport of water molecules from one place to another, we consider a simple system that has two compartments connected by a carbon nanotube (CNT), which was used in our previous work for the study of nonpolar molecule transport [1] (see Figure 1). In contrast to the previous work, in this work, we include 884 water molecules instead of nonpolar molecules. To construct the compartments and nanochannel, we use graphene plates whose *x*–*y* dimensions are 3.03 × 3.22 nm and an armchair (6,6) CNT with a length of 4 nm. To connect the spaces in the compartments and the CNT, we make small holes in the graphene plates next to the CNT. The gap created by the graphene plate and the CNT in Figure 1a is too small for a water molecule to pass through. Figure 1a shows the associated lengths along the *z* axis. To model the graphene plates and the CNT, we use the AMBER force field with ε = 0.3598 kJ/mol and σ = 0.3400 nm for the 6–12 Lennard-Jones (LJ) parameters [11,12]. To model water molecules, we employ TIP3P water [13]. The electrostatic interactions are calculated using the particle-mesh Ewald (PME) method [14]. For LJ interactions, if not specified, the Lorentz-Berthelot rule [15] is used for the LJ parameters, i.e., the sigma (σ) and epsilon (ε) parameters. In this study, the cutoff distance for the electrostatic and LJ interactions is 1.4 nm.

To simulate water transport, we first prepare an initial state of the system in which the water molecules are only in Compartment 1 and not in the CNT or Compartment 2. Then, using the GROMACS package [16], we perform two-step MD simulations with periodic boundary conditions in all three spatial directions and a time step of 2.0 fs for equilibration (see Figure 1b) and transport (see Figure 1c) processes. In the simulations, we fix the positions of the compartments and CNT using the freeze group option of GROMACS [15], while water molecules are free to move. Before the water molecules are allowed to move to the CNT and then to Compartment 2, for the equilibration process, we run an NVT MD simulation for 10 ns at 300 K with a separator, i.e., a molecular barricade, composed of a 6-carbon ring at the boundary between Compartment 1 and the CNT (see the violet hexagons in Figure 1a,b). With the barricade, all water molecules remain in Compartment 1. More details of the system can be found in our previous work [1]. Here, to maintain the temperature, we employ the modified Berendsen algorithm named the V-rescale thermostat [17] with a coupling constant of 0.1 ps, which is implemented in GROMACS. During the simulation, the molecules are equilibrated in Compartment 1. After 10 ns, for the transport process, we remove the barricade and run an NVT MD simulation at 300 K to study the water transport between the two compartments.

To induce water transfer, we use a chemical force created by osmolytes. The osmolyte considered here is a nanoparticle that cannot pass through the CNT and remains in the compartment it was originally placed in. In the presence of osmolytes that interact with water molecules in Compartment 2, the chemical potential in Compartment 2 is lower than that in Compartment 1. Therefore, theoretically, if the chemical potential in Compartment 2 is sufficiently low, water transport to Compartment 2 occurs. To model osmolytes, in this work, we use the same osmolytes that we used in our previous work [1]. The reference osmolyte is a larger-size AMBER carbon atom that has the same LJ epsilon (ε) value as an AMBER carbon atom but a larger LJ sigma (σ) value (0.7 nm) than the AMBER carbon atom (0.34 nm). In our previous work, we demonstrated that the value of 0.7 nm is too large for the reference osmolyte to pass through the CNT while water molecules can, which implies that the CNT can be regarded as a semipermeable membrane in osmosis. 

Since the strength of the interaction between an osmolyte and a water molecule can significantly affect the chemical potential of water in Compartment 2, we prepare various osmolytes by modifying the ε value of the reference osmolyte for the LJ interaction between the oxygen atom of water (TIP3P oxygen atom) and the osmolyte atom. Note that the ε value of the reference osmolyte is 0.47837 kJ/mol. Specifically, we modify the strength by multiplying this reference value by a multiplication factor. We call this multiplication factor the relative water-osmolyte interaction strength (*IS_WO_*). Note that notation *IS_WO_* corresponds to notation *IS_MO_* in our previous work [1]. In this study, we consider *IS_WO_* values of 0.1, 1 (reference), 5, 10, 20 and 50. Figure 1 shows one example of systems containing 50 osmolytes with *IS_WO_* = 10.

To analyze the transfer of water molecules from the water-filled compartment to the water-empty compartment, we calculate the numbers of water molecules residing in Compartment 1 (*N*_1_), the CNT (*N_CNT_*) and Compartment 2 (*N*_2_). One example is shown in Figure 1c. As water transport occurs, *N*_1_ decreases while *N*_2_ increases. *N*_1_ and *N*_2_ show opposite time-transient behaviors, which indicates that the plots of *N*_1_ and *N_CNT_* are sufficient to completely determine *N*_2_. Note that $N_{1}+N_{CNT}+N_{2}=884$ and *N_CNT_* << *N*_1_, *N*_2_ since only a small number of water molecules can occupy the CNT. In this paper, all figures, including Figure 1, are prepared using the VMD [18] (http://www.ks.uiuc.edu/Research/vmd/; University of Illinois at Urbana-Champaign, Urbana, IL, USA) and xmgrace (http://plasma-gate.weizmann.ac.il/Grace/) programs.

## 3. Results and Discussion

### 3.1. Necessary Conditions for Water Transport

#### 3.1.1. General Thermodynamic Considerations

In general, for water transport from Compartment 1 to Compartment 2 to occur, thermodynamic conditions must be created, where the chemical potential of water in Compartment 2 (µ_2_) is lower than that in Compartment 1 (µ_1_), i.e., µ_1_ > µ_2_. This chemical difference induces mass transfer [6]. However, since the two compartments are not directly connected but are connected through a nanochannel, i.e., a CNT, the CNT plays a role. Thus, we require more thermodynamic conditions related to the CNT. In other words, for the transport of water molecules to Compartment 2, water molecules must easily enter the CNT, which implies that the chemical potential of water in the CNT (µ_CNT_) should be less than µ_1_, i.e., µ_1_ > µ_CNT_. Similarly, for water molecules to easily escape from the CNT and enter Compartment 2, the condition µ_CNT_ > µ_2_ must be satisfied. Finally, these conditions can be summarized into one inequality: µ_1_ > µ_CNT_ > µ_2_. Therefore, the key to the transfer of water from Compartment 1 to Compartment 2 is that this inequality holds until all water molecules have moved to Compartment 2. However, since the chemical potential difference decreases as water transport continues, the initial difference should be sufficiently large; when more water molecules move to Compartment 2, the force of osmolytes dragging water molecules from Compartment 1 to Compartment 2 is expected to be reduced because of the increase in number of water molecules surrounding osmolytes in Compartment 2 (screening effect). In the next sections, starting from a reference model in our previous work [1], we individually discuss the necessary conditions for water transport. 

#### 3.1.2. Transport of Water Molecules in the Absence of Osmolytes

As discussed in Section 3.1.1, our model system is based on the model system in our previous work [1] used for the transport of weakly interacting molecules (or charge-removed water molecules). In the previous work, we showed that even in the absence of osmolytes, transport from Compartment 1 to Compartment 2 occurs due to the entropic force (µ_1_ > µ_2_), and at equilibrium (µ_1_ = µ_2_), the number of molecules in each compartment is proportional to the size of the compartment. For example, when Compartment 1 and Compartment 2 have the same size, at equilibrium, the number of molecules in Compartment 1 is the same as that in Compartment 2. To determine if we can observe the same phenomena with water molecules, we use the model system used in our previous work, but we replace charge-removed water molecules with water molecules. 

**Figure 2:**
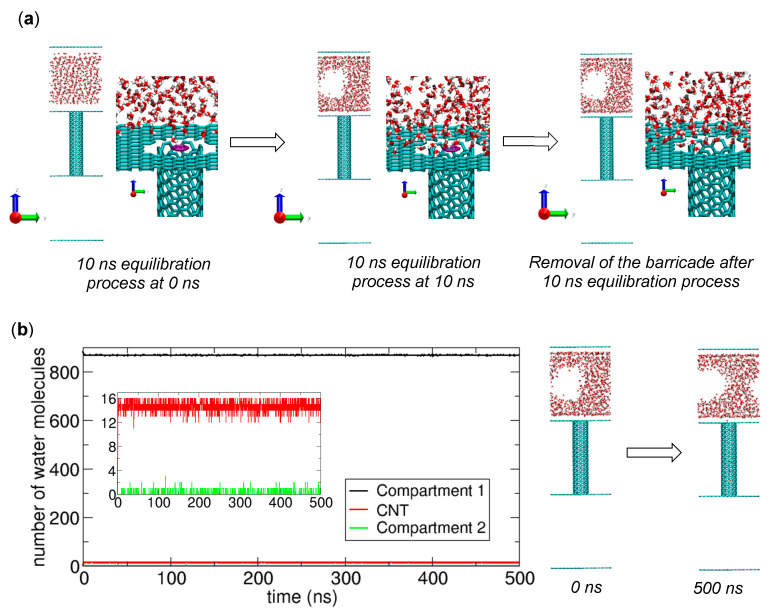
Simulation of water molecule transport from a filled compartment (Compartment 1) to another empty compartment (Compartment 2) through a carbon nanotube (CNT) in the absence of osmolytes. (**a**) Configurations at 0 ns (left) and 10 ns (center) from a 10 ns equilibration process in the presence of a molecular barricade (violet) at the boundary between Compartment 1 and the CNT, and the configuration (right) in which the barricade has been removed, after the equilibration process. The last configuration is used for the simulation of water molecule transport in the next process. (**b**) Results of water transport simulation. Changes in the numbers of water molecules in Compartment 1 (black), the CNT (red), and Compartment 2 (green) during a 500 ns NVT simulation (left) along with snapshots at 0 and 500 ns (right). The inset shows a zoomed-in view of the changes in the CNT and Compartment 2.

The preparation and simulation of systems in the absence of osmolytes are basically the same as those in the presence of osmolytes depicted in Figure 1. Figure 2a shows schematics of the equilibration procedure before a production run. After the 10 ns equilibration, we prepare the initial configuration for the production run and run a 500 ns NVT MD simulation. To determine if transport occurs, we calculate the numbers of water molecules in Compartment 1, the CNT, and Compartment 2 and plot them as functions of time in Figure 2b. The results indicate that transport does not occur, as the number of molecules in Compartment 2 is essentially zero (see the inset of Figure 2b). Apparently, the strong interactions between water molecules prohibit water molecules in Compartment 1 from moving to Compartment 2 because in the absence of such interactions, almost half of the molecules spontaneously move to Compartment 2, as we observed in our previous work [1]. In other words, to initiate water transport, some water molecules in Compartment 1 should dissociate from a group of molecules in Compartment 1 and then transfer to Compartment 2, but since the molecules tend to associate with other water molecules, they tend to remain in Compartment 1, which means that µ_1_ < µ_2_. Therefore, in contrast to the entropy-driven transport of charge-removed water, for water transport, an external force must be used to satisfy µ_1_ > µ_2_. In this work, as an external force, we use an osmotic force created by osmolytes in Compartment 2, which will be discussed in Section 3.1.3.

Notably, the inset of the plot in Figure 2b shows that water molecules occupy the CNT, which occurs because µ_1_ > µ_CNT_ [11]. Another interesting feature from the snapshots in Figure 2b is that a cavity is formed in Compartment 1 and stably maintained, due to the attractive interaction between the wall of the compartment and water molecules. Previously, the related hydrophilicity of graphene plates was discussed [19]. One may think that this cavity is compatible with the water transport through the CNT, but it can lead to early termination of the transport process before a majority of water molecules are transferred to Compartment 2, which will be explained in Section 3.1.4.

#### 3.1.3. Transport of Water Molecules in the Presence of Osmolytes

**Figure 3:**
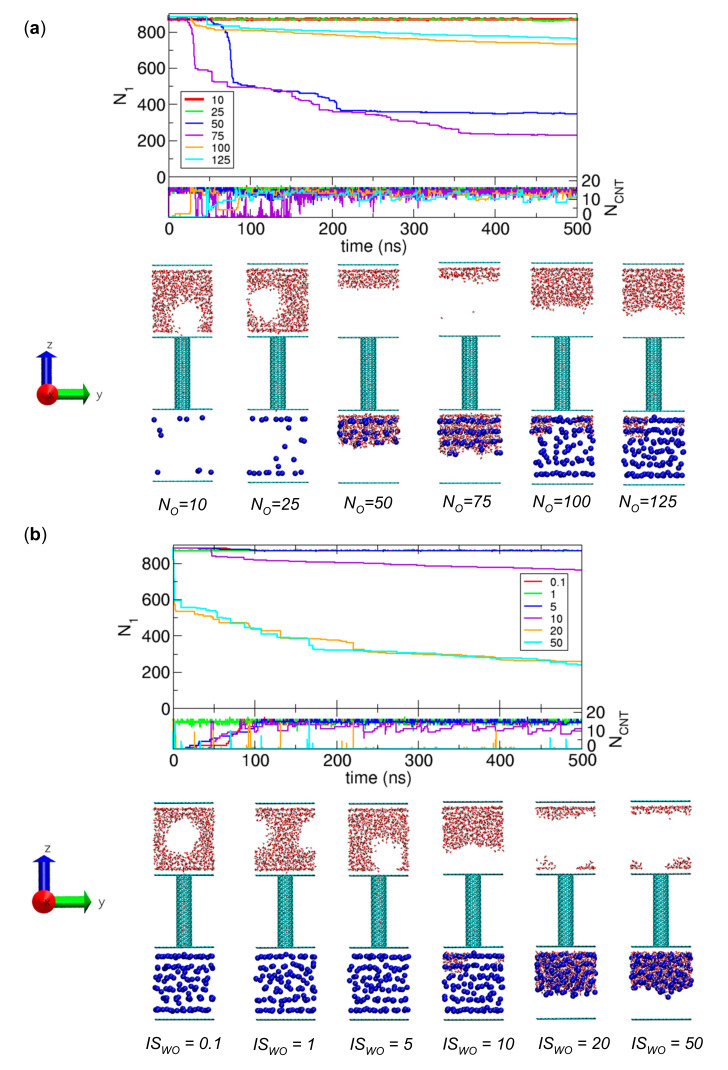
Influence of osmolytes on water transport. (**a**) Numbers of water molecules in Compartment 1 (*N*_1_) and the CNT (*N_CNT_*) as functions of time for the cases with *IS_WO_* = 10 and *N_O_* = 10 (red), 25 (green), 50 (blue), 75 (violet), 100 (orange), and 125 (cyan), and their final configurations at 500 ns. (**b**) Numbers of water molecules in Compartment 1 (*N*_1_) and the CNT (*N_CNT_*) as functions of time for the cases with *N_O_* = 125 and *IS_WO_* = 0.1 (red), 1 (green), 5 (blue), 10 (violet), 20 (orange), and 50 (cyan).

As discussed in Section 3.1.2, without osmolytes, the chemical potential of water in Compartment 2 is higher than that in Compartment 1: µ_1_ < µ_2_. Therefore, to create a transport-inducible environment satisfying µ_1_ > µ_2_, we can place osmolytes in Compartment 2. A sufficient number of osmolytes and a sufficient strength of the interaction with water molecules will induce water transport. To obtain a general idea of the effect of osmolytes, we examine some representative cases with various numbers of osmolytes (*N_O_*) and various strengths of the interaction (*IS_WO_*). The simulation results for these cases are presented in Figure 3. 

In Figure 3a, we fix the value of *IS_WO_* as 10 and consider several values of *N_O_*: *N_O_* = 10, 25, 50, 75, 100, and 125. These specific values of *N_O_* are selected because they were used for the transport study of charge-removed water molecules in our previous work [1], and it is necessary to use the same values in this work for the comparison to understand the effect of the interaction strength between transported molecules, as will be discussed in Section 3.2.5. From the simulations, we observe water transport for the cases of *N_O_* = 50, 75, 100, and 125. One may expect that as *N_O_* increases, more water molecules would be transferred to Compartment 2, but surprisingly, we obtain a different result in that the numbers of water molecules in Compartment 2 for the cases of *N_O_* = 50 and 75 are larger than those for the cases of *N_O_* = 100 and 125. This is related to the cavity initially formed in Compartment 1 due to the compartment-water interaction. During transport, the cavity readily breaks a group of water molecules into two disconnected groups: one group is next to the lower graphene plate and CNT, and the other group is next to the upper graphene plate. The water molecules in the former group transfer to the CNT but the water molecules in the latter group remain in Compartment 1. Therefore, the cases of *N_O_* = 100 and 125 have fewer transferred water molecules because the numbers of residual water molecules in the latter group are greater than those in the cases of *N_O_* = 50 and 75.

In contrast to Figure 3a, in Figure 3b, we fix the value of *N_O_* as 125 and consider various values of *IS_WO_*, *IS_WO_* = 0.1, 1, 5, 10, 20, and 50. These specific values of *IS_WO_* were used in our previous work with charge-removed water molecules [1]. In this case, when *IS_WO_* = 10, 20, and 50, we observe water transport to Compartment 2. However, because of the breakage due to the cavity during transport, we also observe residual water molecules next to the upper graphene plate of Compartment 1, as shown in Figure 3a. Additionally, we note that because of the strong interaction with *IS_WO_* = 20 and 50, all water molecules in the CNT are transferred to Compartment 2, and thus, the CNT is empty; presumably, in this case, µ_CNT_ >> µ_2_.

#### 3.1.4. Transport of Water Molecules in Compartments that Weakly Interact with Water

**Figure 4:**
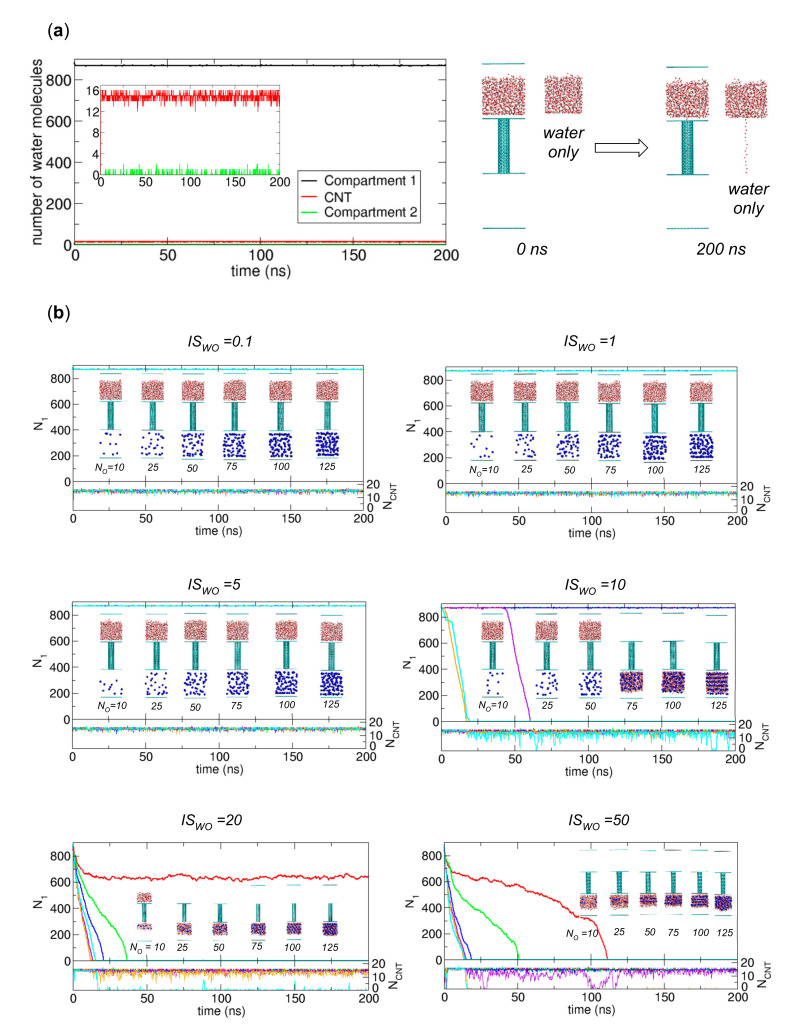
Simulation of water molecule transport in the systems composed of compartment walls that weakly interact with water molecules in the (**a**) absence and (**b**) presence of osmolytes with *N_O_* = 10 (red), 25 (green), 50 (blue), 75 (violet), 100 (orange), and 125 (cyan) and *IS_WO_* = 0.1, 1, 5, 10, 20, and 50. For each system, the numbers of water molecules in Compartment 1 (*N*_1_) and the CNT (*N_CNT_*) are plotted as functions of time. The insets show the final configurations of systems at 200 ns. Note that for all cases, the cavities in Figure 2 and Figure 3 disappear.

From the discussion in Section 3.1.3, we clearly must remove the cavity in Compartment 1 to transport more water molecules to Compartment 2. Since the cavity is created because of the attraction between the compartment wall (normal graphene plates) and water molecules, one method to remove the cavity is to weaken the compartment-water interaction. To implement this idea, we reduce the LJ interaction between carbon atoms in the graphene plates and water molecules via the Lorentz-Berthelot rule [15], while the interaction between carbon atoms in the CNT and water molecules remains the same as in the original system. Specifically, we reduce the standard value of ε for carbon, i.e., 0.3598 kJ/mol, by 10 times, so ε = 0.03598 kJ/mol. We expect that this reduction guarantees the condition µ_1_ > µ_CNT_. With this weak compartment-wall interaction, we perform MD simulations and examine extensive cases with *N_O_*=10, 25, 50, 75, 100, and 125 and *IS_WO_* = 0.1, 1, 5, 10, 20 and 50 to find the necessary osmolyte conditions for water transport. The simulation results are displayed in Figure 4, which is the main figure of this work. 

Before we study the effect of osmolytes for the new systems above, we run a simulation for the system in the absence of osmolytes to understand the effect due solely to the weak interactions between compartments and water molecules. The configurations in Figure 4a show that the weak interactions remove the cavity in Compartment 1, and the water molecules in Compartment 1 are next to the lower graphene plate, not the upper graphene plate. This occurs because in Compartment 1 the water-water and water-CNT interactions are stronger than the reduced water-graphene interactions. Additionally, because of the reduction in the compartment-water interaction, water molecules are less likely to move from the CNT to Compartment 2, i.e., µ_CNT_ < µ_2_. Therefore, as shown in Figure 4a, water molecules remain in Compartment 1 and the CNT; thus, in this case, osmolytes are required to induce water transport to Compartment 2, as shown in Section 3.1.3. 

When we place osmolytes in Compartment 2 and weaken the compartment-water interactions, we observe complete or nearly complete water transport in some cases in the sense that all water molecules are removed from Compartment 1 and transferred to CNT or Compartment 2. In these cases, the osmotic effect is significant. From Figure 4b, the cases showing (nearly) complete water transfer are the cases with *N_O_* = 75, 100, and 125 for *IS_WO_* = 10, *N_O_* = 25, 50, 75, 100, and 125 for *IS_WO_* = 20, and *N_O_* = 10, 25, 50, 75, 100, and 125 for *IS_WO_* = 50. One exceptional case is that with *N_O_* = 10 and *IS_WO_* = 20 in that only some of the water molecules in Compartment 1 are transferred, and some residual water molecules remain in Compartment 1. Including this exceptional case, we later analyze all the cases in terms of kinetically stable states. 

**Figure 5:**
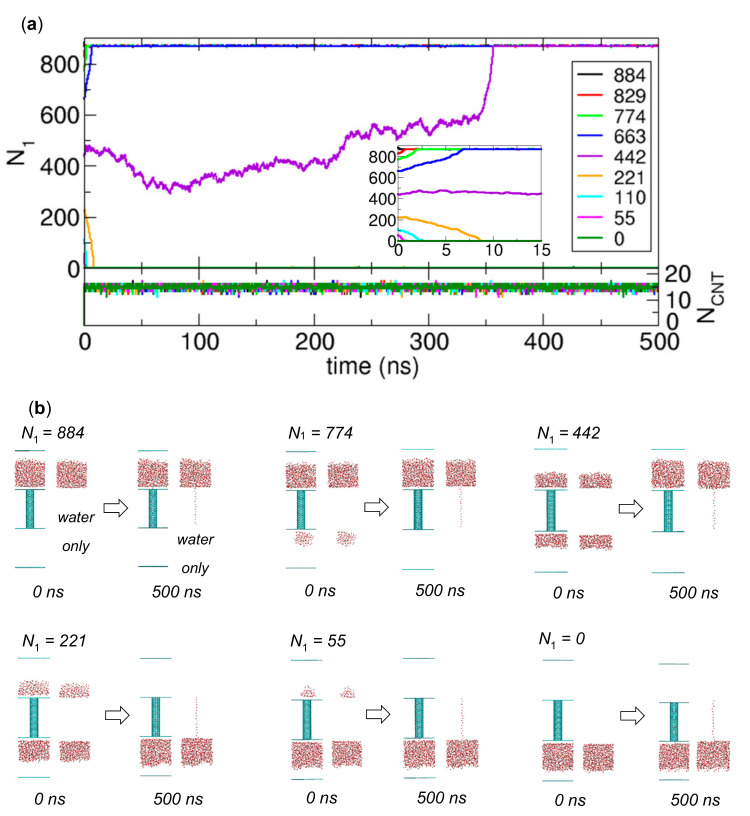
Transport simulation of water molecules in various systems of compartment walls that weakly interact with water molecules in the absence of osmolytes. Various initial configurations with *N*_1_ = 884, 829, 774, 663, 442, 221, 110, 55 and 0 are prepared from the 10 ns equilibration process. (**a**) Numbers of water molecules in Compartment 1 (*N*_1_) and the CNT (*N_CNT_*) as functions of time. The inset show the plots for a time interval from 0 ns to 15 ns. (**b**) Initial and final configurations obtained from the 500 ns MD simulations for various initial configurations with *N*_1_ = 884, 774, 442, 221, 55 and 0.

Additionally, we consider the possibility of water transport between the compartments without osmolytes. To address this possibility, it is the best to discuss with a free energy profile of system as a function of *N*_1_ or *N*_2_, but it is generally challenging to obtain an accurate free energy profile, and it requires a lot of computer resources. Therefore, to obtain a general idea of the shape of the free energy profile, we prepare various systems, which represent intermediate states that can be observed in the water transport, by preparing the initial configurations with *N*_1_ = 884, 829, 774, 663, 442, 221, 110, 55 and 0. Then, we perform the MD simulations to observe the time evolution of states. The simulation results are summarized in Figure 5. Figure 5a indicates that the intermediate states go to either the state where water molecules occupy only Compartment 1 and CNT (State A; the cases with *N*_1_ = 884, 829, 774, 663, and 442) or the state where they occupy only Compartment 2 and CNT (State B; the cases with *N*_1_ = 221, 110, 55, and 0). The final configurations in Figure 5b clearly show that there are only these two stable states. From Figure 5a, we also observe that initially when *N*_1_ > *N*_2_ or *N*_2_ > *N*_1_, the initial state quickly goes to State A or State B, respectively. However, initially when *N*_1_ = *N*_2,_ *N*_1_ is largely fluctuating before the state eventually goes to State A or State B; in fact, this fluctuation is similar to the one observed when the two groups pull against each other at opposite ends of a rope with similar strength in a tug of war. Based on the results in Figure 5, we expect that free energy profile has the minima near *N*_1_ = 884 (State A) and 0 (State B) and a higher value (possibly the maximum) near *N*_1_ = 442, the slope near the minima is relatively large, and the slope near *N*_1_ = 442 is small, which enables a large fluctuation in *N*_1_. This shape of free energy profile implies that there is a free energy barrier in the transition between stable States A and B. Thus, we need an external force such as osmotic pressure to induce water transport. For charge-removed water molecules [1], the free energy profile is expected to show the opposite behavior with the minimum near *N*_1_ = 442; therefore, it is interesting to see how the free energy profile changes with the strength of interaction among transported molecules, which will be a subject of future study. 

### 3.2. Water Transport Analysis

#### 3.2.1. Potential Energy Change

In Section 3.1.4, we observe water transport from the 200 ns simulations. Now, the following question arises: what is the driving force for the water transport? Since this is an NVT simulation, the appropriate free energy for the system is the Helmholtz free energy A=E−TS, where E, T, and S are the internal energy, temperature, and entropy, respectively [6]. Therefore, to systematically understand the Helmholtz free energy change Δ*A*, we can consider the energetic contribution related to the first term E and the entropic contribution related to the second term TS. However, from Figure 2 and Figure 3, we understand that the energetic contribution is dominant over the entropic contribution because if the entropic change is dominant, water molecules should be transferred to Compartment 2 due to the translational entropy and the mixing entropy with osmolytes. Therefore, we focus on the internal energy change Δ*E*, which is the sum of the potential energy change and kinetic energy change. Furthermore, since we study the system under a constant temperature, we expect that the change due to the kinetic energy is not significant. Thus, we expect that the potential energy change is responsible for the water transport. We calculate the potential energy change to determine if the change is correlated with the water transport, to explain the driving force for the transport. 

**Figure 6:**
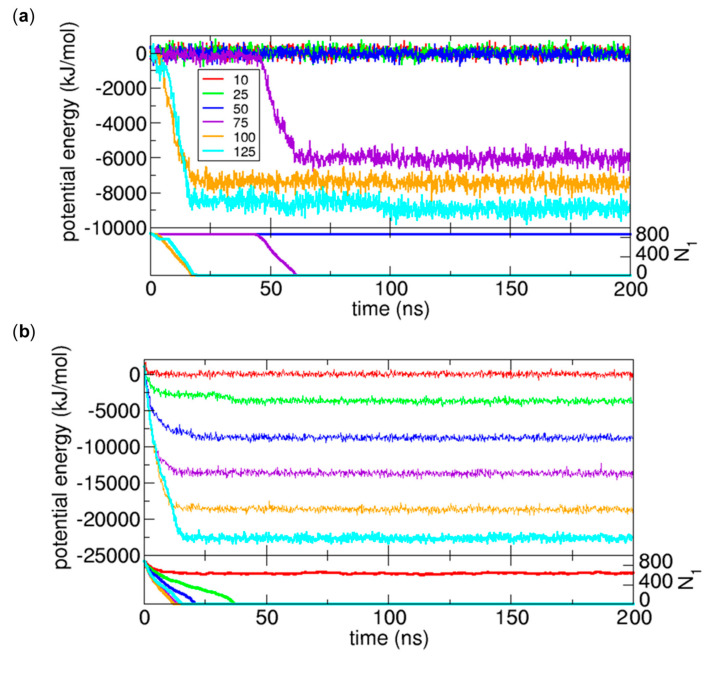

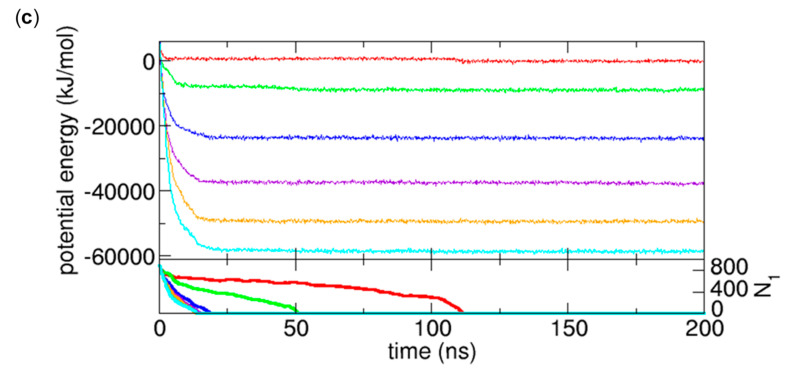
Changes in potential energy and in the number of water molecules in Compartment 1 (*N*_1_) as functions of time for the cases of *N_O_* = 10, 25, 50, 75, 100, and 125 when (**a**) *IS_WO_* = 10 (**b**) *IS_WO_* = 20, and (**c**) *IS_WO_* = 50.

As shown in Figure 6, we calculate the potential energy change for the cases of *IS_WO_* = 10, 20, and 50, where water transport is observed. In this calculation, for convenience, for each value of *IS_WO_*, we set the average potential energy over the time interval between 100 ns and 200 ns for the *N_O_* = 10 case to zero. The potential energies for the other cases are calculated based on this reference. As we expect, when water transport occurs, the potential energy change is significant and is highly correlated with the change in *N*_1_, which implies that the potential energy change due to the mixing of water molecules and osmolytes is the major driving force for water transport driven by osmosis. 

#### 3.2.2. Kinetically Stable States 

From Figure 4, we observe kinetically stable states in terms of the numbers of water molecules in Compartment 1, the CNT and Compartment 2. Specifically, we find three main stable states, some of which, we believe, are global equilibrium states. In other words, the initial state in which water molecules are only in Compartment 1 transitions to one of three states: in one state, water occupies only Compartment 1 and the CNT (State I); in another state, water occupies only the CNT and Compartment 2 (State II); and in the other state, water occupies only Compartment 2 (State III). Here, State I can be considered a metastable state in the presence of osmolytes. Specifically, when we compare a separated state in which water molecules are in Compartment 1 and osmolytes are in Compartment 2 (State I) with a mixed state in which water molecules and osmolytes are in Compartment 2 (State II and State III), the mixed state is more energetically favorable than State I because of the strong interaction between water molecules and osmolytes. Therefore, the observation of State I is probably due to the kinetic barrier for the transition to State II or State III. In Figure 4, the only exception to the above three states is the stable state in the case of *N_O_* = 10 and *IS_WO_* = 20, in which water molecules exist in all regions. We will discuss this state later, which we believe is another metastable state. 

When the interaction between osmolytes and water molecules is weak, i.e., *IS_WO_* = 0.1, 1, and 5, the systems reach State I, and transfer to Compartment 2 does not occur. Additionally, when the interaction is intermediate, i.e., *IS_WO_* = 10, and the number of osmolytes is not sufficient to induce water transport (*N_O_* = 10, 25, and 50), the system also reaches State I. However, when *N_O_* = 75, 100, and 125, the stable states are State II. Moreover, when the interaction is strong, i.e., *IS_WO_* = 20, 50, even a small number of osmolytes can induce water transport and the states also become State II. Specifically, the cases with *N_O_* = 25, 50, 75, and 100 for *IS_WO_* = 20 and *N_O_* = 10, 25, 50, and 75 for *IS_WO_* = 50 show State II as a stable state. Here, interestingly, the addition of more osmolytes induces complete transfer of water molecules to Compartment 2, which means that the stable states are State III. Specifically, the cases with *N_O_* = 125 for *IS_WO_* = 20 and *N_O_* = 100 and 125 for *IS_WO_* = 50 show State III as a stable state. The observations for the stable states can be summarized in Table 1. 

Table 1 shows that when the interaction strength *IS_WO_* and number of osmolytes *N_O_* increase, the kinetically stable state shifts from State I to State II to State III. For example, from the right-most column (*N_O_* = 125) in Table 1, we easily note that State I, State II, and State III appear in order when *IS_WO_* increases. Similarly, if we examine the states along the row of *IS_WO_* = 10 (the states with the same *IS_WO_* value of 10) in the table, we find that as *N_O_* increases, State I and State II appear in order. Here, one interesting question is whether we can observe State III if we increase the number of osmolytes. To address this question, we could insert more osmolytes into Compartment 2. However, since the compartment space is limited in terms of the number of osmolytes, we use an alternative method in which we increase the ratio of the number of osmolytes to the number of water molecules (*N_O_*/*N_total_*) by reducing the total number of water molecules instead of increasing the number of osmolytes. 

**Figure 7:**
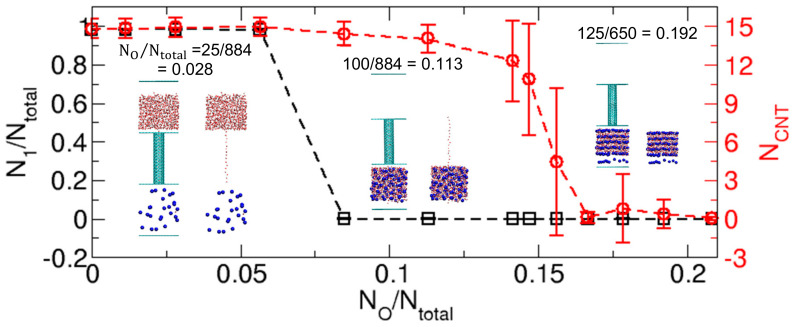
Kinetically stable states observed for a range of *N_O_*/*N_total_*, the ratio of the number of osmolytes (*N_O_*) to the total number of water molecules (*N_total_*). The stable states are characterized by the occupancy ratio of water molecules in Compartment 1 (*N*_1_/*N_total_*) and the number of water molecules in the CNT (*N_CNT_*), averaged over the time interval from 100 ns to 200 ns in the 200 ns simulations. The insets illustrate three representative configurations of States I, II, and III, whose values of *N_O_*/*N_total_* are 0.028, 0.113, and 0.192, respectively, which are taken at 200 ns from the 200 ns simulations. The error bars indicate the standard deviations.

To determine if higher ratios of *N_O_*/*N_total_* in the cases of *IS_WO_* = 10 induce State III, we prepare systems with fewer water molecules based on the system of *N_O_* = 125, *IS_WO_* = 10 and *N_total_* = 884 (*N_O_*/*N_total_* = 0.141). In other words, from the final state at 200 ns shown in Figure 4, we remove some of the water molecules, so that *N_total_* = 850, 800, 750, 700, 650, and 600, which implies that *N_O_*/*N_total_* = 0.147, 0.156, 0.167, 0.179, 0192, and 0.208, respectively. From the 200 ns simulations of the systems, we calculate the occupancy ratio of water in Compartment 1 (*N*_1_/*N_total_*) and the number of water molecules in the CNT (*N_CNT_*). With the cases of *N_total_* =884 in Figure 4, we summarize the simulation results as a function of *N_O_*/*N_total_* in Figure 7. The results clearly indicate that at high values of *N_O_*/*N_total_* (>~0.17), the stable states are State III. In other words, *N*_1_/*N_total_* and *N_CNT_* are practically zero. This result may suggest that in the original system of 884 water molecules, if we include more osmolytes, i.e., the ratio of *N_O_*/*N_total_* increases, we will observe State III as a stable state. 

Another interesting feature from Figure 7 is that the number of water molecules in the CNT largely fluctuates in the transition regime between State II and State III, whose *N_O_*/*N_total_* value is approximately 0.15. This fluctuation in *N_CNT_* is a typical behavior observed in the wetting-dewetting transition [20].

One remaining issue that we have to address is whether the state observed in the case of *N_O_* = 10 and *IS_WO_* = 20 is a global equilibrium state or a metastable state. Here, a metastable state means that the state eventually transitions to another more stable state if we wait. Considering the stable states observed for other values of *N_O_* (see the row of *IS_WO_* = 20 in Table 1), one would guess that State II is a more stable (equilibrium) state. 

**Figure 8:**
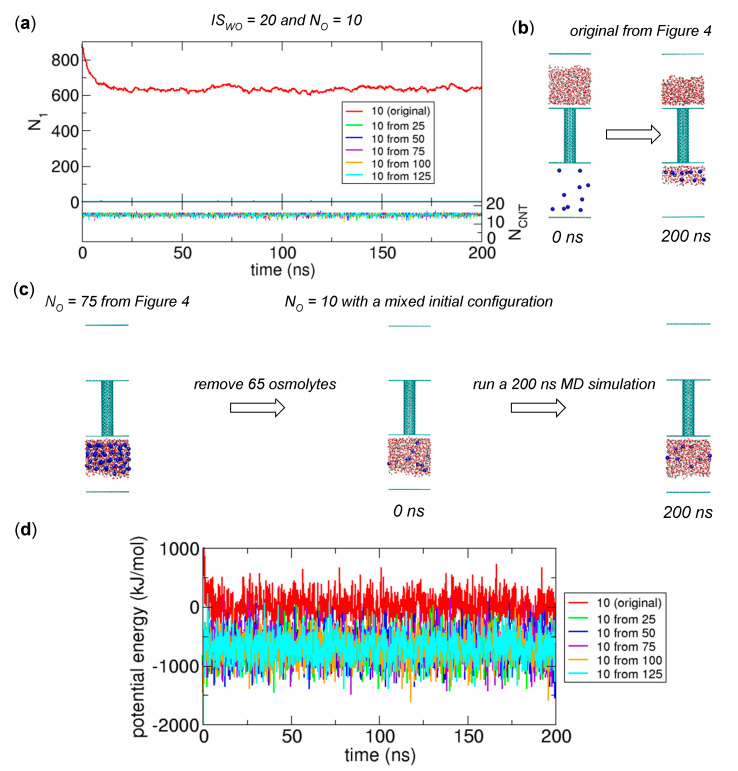
Equilibrium states for the case of *N_O_* = 10 and *IS_WO_* = 20 from the MD simulations with mixed initial configurations. The mixed initial configurations are prepared by removing some osmolytes from the final configurations at 200 ns in Figure 4 for the cases of *N_O_* = 25, 50, 75, 100, and 125 and *IS_WO_* = 20. (**a**) Numbers of water molecules in Compartment 1 (*N*_1_) and the CNT (*N_CNT_*) as functions of time for the cases with the original initial configuration (red) and mixed configurations (other colors). (**b**) Original initial configuration and final configuration at 200 ns obtained from the simulations in Figure 4. (**c**) Mixed initial configuration prepared from the final configuration with *N_O_* = 75 at 200 ns in Figure 4. (**d**) Changes in potential energy as functions of time. In this plot, the average value of the potential energy for the original case over the time interval from 100 to 200 ns is set to zero.

To examine whether State II is a more stable state for the case of *N_O_* = 10 and *IS_WO_* = 20, we first prepare five such initial states by removing some osmolytes from the final states (State II) with *N_O_* = 25, 50, 75, 100, and 125 and *IS_WO_* = 20 at 200 ns in Figure 4, and run MD simulations to determine whether the states characterized as State II are stable. The steady state in terms of *N*_1_ and *N_CNT_* in Figure 8a indicates that State II is kinetically stable. The relevant configurations for the originally observed state and State II are displayed in Figure 8b,c, respectively. Then we compare the potential energies between the original state and State II, as displayed in Figure 8d. From the comparison, we see that the potential energies of State II are lower than the potential energy of the original state observed in Figure 4. In particular, since the potential energy difference (~700 kJ/mol on average) is significant, we conclude that the stable state in Figure 4 is a metastable state, and State II is a global equilibrium state. The physical reason for the stability in the metastable state is probably associated with the state being stuck in a local energy minimum, which implies that for further investigations, we may need to examine the spatial arrangements of osmolytes and water molecules in detail. 

Finally, if we consider the water transport process as a process to reach an equilibrium state, we can regard the water transport as a transition process from the initial state to State II or State III. In particular, we can call the transition to State III complete transfer of water because all water molecules are transferred from Compartment 1 to Compartment 2 due to the osmolytes.

#### 3.2.3. Stochastic Nature of Water Transport Occurrence

Figure 4 shows that water transport occurs at a very early stage of the simulations when *IS_WO_* is large (20 or 50), while transport does not occur when *IS_WO_* is small (0.1, 1, or 5). However, when *IS_WO_* is intermediate (10), water transport can occur or not depending on the number of osmolytes *N_O_*; in Figure 4, it occurs only when *N_O_* is greater than 75.

**Figure 9:**
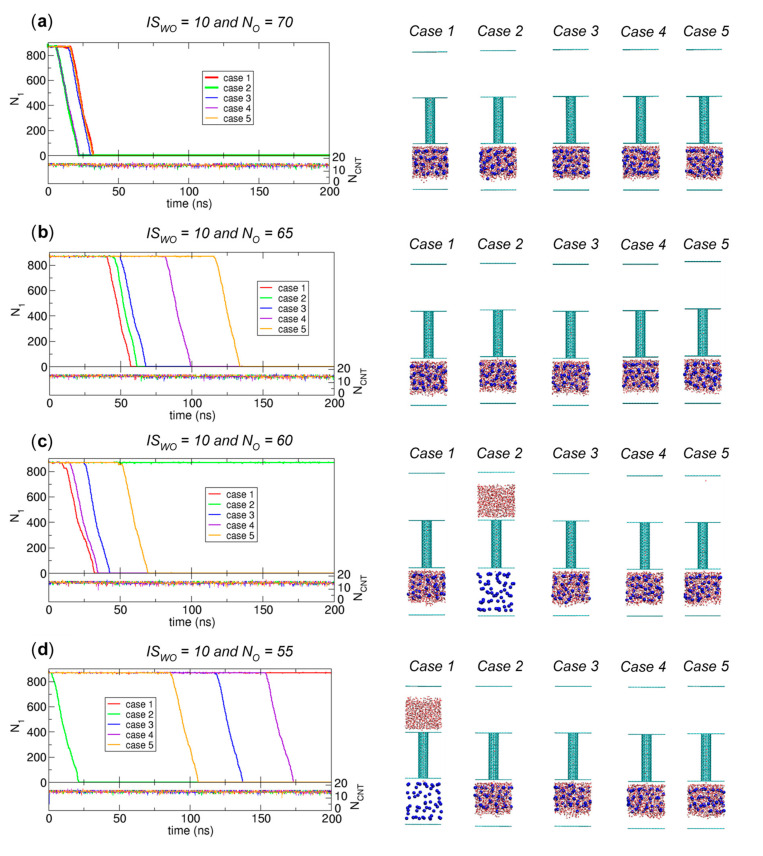

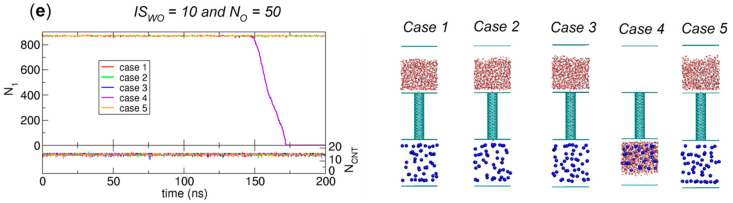
Results of 200 ns simulations for the systems of *N_O_* = (**a**) 70, (**b**) 65, (**c**) 60, (**d**) 55, and (**e**) 50 with *IS_WO_* = 10. For each system, we perform five independent simulations, and we plot the numbers of water molecules in Compartment 1 (*N*_1_) and the CNT (*N_CNT_*) as functions of time (left) with the final configurations at 200 ns from the five simulations (right).

For *IS_WO_* = 10, to better understand the *N_O_* criterion for the occurrence of water transport, we examine more systems between the two systems with *N_O_* = 75 (transport observed) and 50 (no transport) in Figure 4, which correspond to *N_O_* = 70, 65, 60, 55, and 50. For each system, we perform five independent simulations. The simulation results are shown in Figure 9. 

Interestingly, for the systems of *N_O_* = 65, 60, and 55, the occurrence clearly shows a stochastic nature, which means that simulations with a given *N_O_* can exhibit water transport or not, and moreover, in the cases where water transport occurs, the waiting time for water transport to occur could vary. For example, when *N_O_* = 55, four cases of five display water transport, and their waiting times are 0.2 ns (Case 2), 117.8 ns (Case 3), 153.8 ns (Case 4), and 86.4 ns (Case 5).

For *N_O_* = 70, however, we observe water transport in all five cases, and the waiting times are relatively narrowly distributed (16.2 ns (Case 1), 6.0 ns (Case 2), 14.2 ns (Case 3), 6.2 ns (Case 4), and 16.0 ns (Case 5)). In contrast, for the cases of *N_O_* = 50, in only one case (Case 4) is water transport observed. From this comparison, as *N_O_* decreases, the waiting time increases in that the waiting time is at least 200 ns for the case with no transport. The observation with *N_O_* = 50 gives rise to a question: if we consider a longer simulation time, such as 1000 ns, than the value of 200 ns in Figure 9, can we observe more cases showing water transport? To address this question, we further examine longer simulations for more cases in which one might expect no water transport from the 200 ns simulation results in Figure 4. 

**Figure 10:**
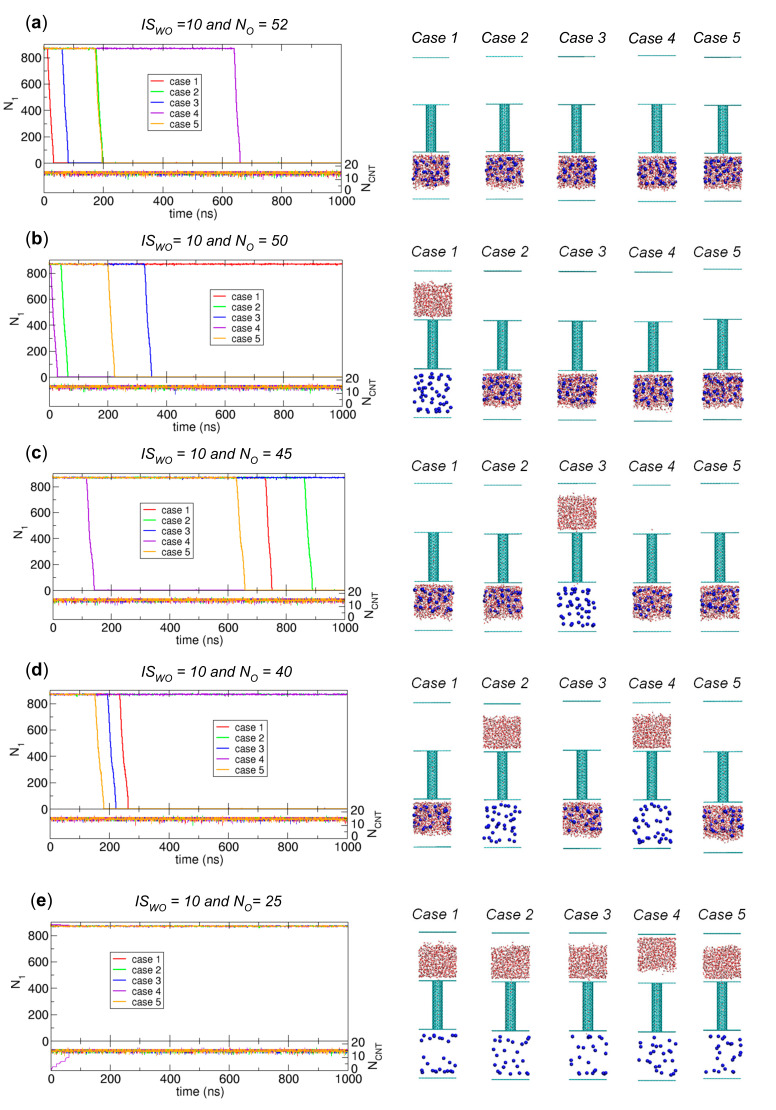
Results of 1000 ns simulations for the systems of *N_O_* = (**a**) 52, (**b**) 50, (**c**) 45, (**d**) 40, and (**e**) 25 with *IS_WO_* = 10. For each system, we perform five independent simulations, and we plot the numbers of water molecules in Compartment 1 (*N*_1_) and the CNT (*N_CNT_*) as functions of time (left) along with the final configurations at 1000 ns from the five simulations (right).

We perform 1000 ns simulations for *N_O_* = 52, 50, 45, 40, and 25. For each *N_O_*, we perform five independent simulations. We display the simulation results in Figure 10. The simulation results for *N_O_* = 50 and 45 indicate that up to 200 ns, only two cases of five and only one case exhibit water transport, respectively, but up to 1000 ns, for each *N_O_*, four cases of five show water transport. Therefore, when we increase the simulation time, the probability for water transport increases. Figure 10 also shows that when *N_O_* decreases, the number of water transport occurrences is reduced: five, four, four, three, and zero occurrences for *N_O_* = 52, 50, 45, 40, and 25, respectively. Therefore, from the above discussion, we see the general trend that water transport is more likely to be observed when we include more osmolytes and wait longer; moreover, the waiting time is reduced when more osmolytes are added. Thus, although we do not observe water transport for *N_O_* = 25 in Figure 10, its observation may be possible if we wait much longer.

Finally, related to the discussion of kinetically stable states in Figure 7, since the CNT is occupied by water at all times in Figure 9 and Figure 10, the transition due to the water transport in Figure 9 and Figure 10 is from the initial state to State II. Figure 10 also shows that only the system of *N_O_* = 25, which corresponds to *N_O_*/*N_total_* = 0.028 (= 25/884), does not show water transport. Therefore, to make Figure 7 more accurate, we must adjust the *N_O_*/*N_total_* criterion value to distinguish between States I and II. However, since determining the exact value of *N_O_*/*N_total_* for the boundary requires large-size ensembles and long-time simulations, which is not our major interest, we do not attempt to update Figure 7 based on the results in Figure 9 and Figure 10. Again, the main point of Figure 7 is that mainly three kinetically stable states are observed.

#### 3.2.4. Characteristics of the Transition due to Water Transport 

**Figure 11:**
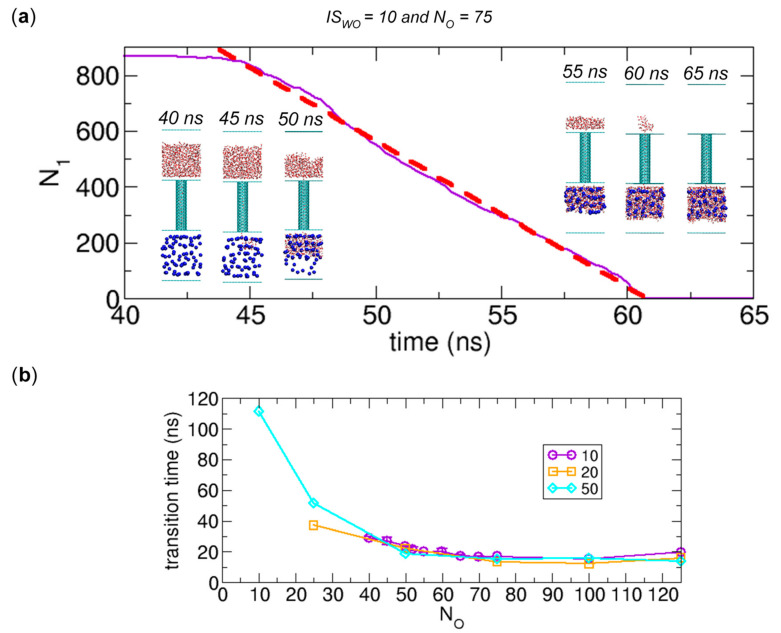

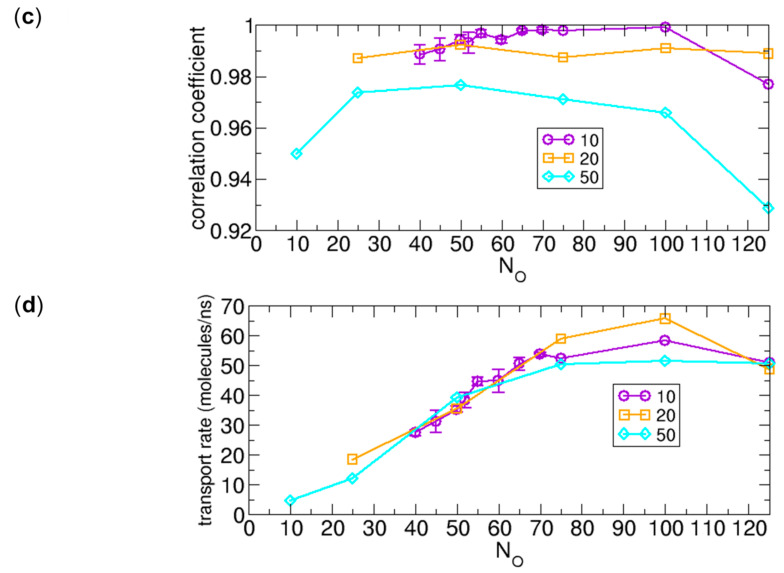
Detailed analysis of water transport observed in Figure 4, Figure 9, and Figure 10. (**a**) Linear curve fitting for the data of *N*_1_ versus time *t* (ns). The curve fitting is applied only to the water transport region. The resulting linear curve is $N_{1}=-52.3t+3179.6$ and the correlation coefficient is 0.998. The configurations in the insets are provided to show what occurs during water transport. (**b**) Transition time from the initial state to State II or State III due to water transport (or duration of water transport) as functions of the number of osmolytes (*N_O_*) for *IS_WO_* = 10, 20, and 50. (**c**) Correlation coefficient calculated from the linear curve fitting as a function of *N_O_* for *IS_WO_* = 10, 20, and 50. (**d**) Transport rate obtained from the slopes of the fitted linear curves as a function of *N_O_* for *IS_WO_* = 10, 20, and 50. When multiple data sets for water transport are available for one system (e.g., *N_O_* = 50 and *IS_WO_* = 10 in Figure 9 and Figure 10), we calculate the average over the multiple sets and the associated standard deviation and plot them in the figures.

Here, we further study the details of water transport observed in Figure 4, Figure 9 and Figure 10. Interestingly, during water transport, the number of water molecules in Compartment 1 appears to linearly decrease with time. To quantify this time-transient behavior, we fit the data to a linear curve. One example is shown in Figure 11a for the case of *N_O_* = 75 and *IS_WO_* = 10 (also see Figure 4), where the data of *N*_1_ versus time *t* are best fitted with a linear curve using curve fitting in the xmgrace program. The resulting linear curve is $N_{1}=-52.3t+3179.6$. This fitting is only valid for the time interval corresponding to the water transport. Remarkably, in this case, the correlation coefficient is greater than 0.99. Moreover, we calculate the transition time, which is the time for the transition from the initial state to State II or State III due to water transport. In this case, the transition time is 17.0 ns (= 60.8 ns − 43.8 ns). Additionally, from the linear fitting, we can determine the slope, which gives the transport rate. In this case, the transport rate is 52.3 water molecules/ns. 

Since the transition time, linearity of the time-transient behavior, and transport rate can depend on the number of osmolytes *N_O_*, we calculate them as functions of *N_O_*. The results are shown in Figure 11. Interestingly, according to Figure 11b, when the number of osmolytes is sufficiently large (*N_O_* > ~60), the transition time is ~ 20 ns almost regardless of the interaction strength *IS_WO_*. Thus, if the osmotic force is sufficiently large beyond a certain value, the transition time reaches a limit, which is ~20 ns in this case. However, more osmolytes can reduce the waiting time for water transport, as we discuss in 3.2.3. When the number of osmolytes is smaller (*N_O_* < ~60), the transition time depends on both *N_O_* and *IS_WO_*. In other words, when the osmotic force decreases by reducing *N_O_* or *IS_WO_*, the transition time increases, or no transition is observed. 

In Figure 11c, we quantitatively analyze the linearity of the time-transient behavior in water transport by calculating the correlation coefficient from the linear curve fitting to the data of *N*_1_ versus time *t*. Surprisingly, when *IS_WO_* = 10, the correlation coefficient is very close to 1, which implies a constant flow of water during the transport. However, as *IS_WO_* increases or when *N_O_* is large, the transient behavior slightly deviates from linearity. This may reflect the nonlinear nature of interactions between molecules involved in water transport. 

As shown in Figure 11d, we calculate the transport rate from the slope obtained from the linear fitting shown in Figure 11a. Noticeably, the three plots for *IS_WO_* = 10, 20, and 50 are similar, and the common features are that when *N_O_* > ~60, the transport rate is almost a constant value (~53 water molecules/ns) with small variations and when *N_O_* < ~60, it decreases as *N_O_* decreases. Note that the plots in Figure 11b,d are inversely related to each other. The similarity of the transport rates regardless of *IS_WO_* for the region of ~40 < *N_O_* < ~60 is an interesting feature, but to better understand its physical origin, further detailed analysis may be required, which is beyond the scope of this work.

#### 3.2.5. Effect of Interactions between Water Molecules on Transport

**Figure 12:**
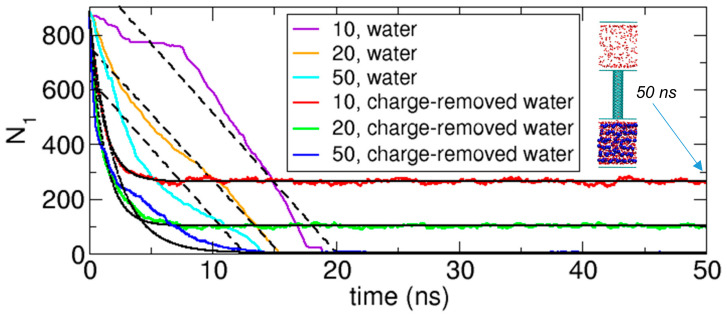
Comparison of the transport of water molecules with the transport of charge-removed water molecules [1] in terms of the numbers of molecules in Compartment 1 (*N*_1_) as functions of time for the cases of *N_O_* =125 and *IS_WO_* = 10, 20, and 50. The black lines represent the linear fitting for the transport of water molecules and the exponential fitting for the transport of charge-removed water molecules. The inset figure shows the equilibrium state at 50 ns for the charge-removed water case of *N_O_* = 125 and *IS_WO_* = 10.

To understand how the interaction between water molecules affects the transport through a nanochannel, we compare the transport of water molecules with the transport of charge-removed water molecules in terms of the number of molecules in Compartment 1 as a function of time. The results are displayed in Figure 12. Here, water and charge-removed water molecules represent strongly interacting and weakly interacting transported molecules, respectively. Charge-removed water molecules are prepared by removing the electric charges of water molecules or setting the electric charges to zero. The transport of charge-removed water molecules was discussed in detail in our previous work [1].

In Figure 12, first, we can compare the cases of water with the cases of charge-removed water in terms of equilibrium states. At equilibrium, while the number of molecules in Compartment 1 (*N*_1_) for water is zero, *N*_1_ for charge-removed water is not zero when *IS_WO_* = 10 and 20; only when *IS_WO_* = 50 does *N*_1_ fluctuate near zero. Without electrostatic interactions, the interactions between charge-removed water molecules are weak, and thus, their physical behavior is similar to that of a gas. Therefore, if the interaction between molecules and osmolytes is not sufficiently strong, then the molecules can fill up the space, and as a result, when *IS_WO_* = 10 and 20, some water molecules remain in Compartment 1 (see the inset of Figure 12). When *IS_WO_* increases, *N*_1_ is reduced, and especially when *IS_WO_* = 50, *N*_1_ is near zero. However, for water, because of the strong interactions, its behavior is liquid-like, and aggregation of water molecules is energetically favorable. Moreover, because of the osmolytes in Compartment 2, being in Compartment 2 is more favorable than being in Compartment 1 for water molecules. Therefore, combining these factors, we understand that water molecules transfer to Compartment 2, and thus, *N*_1_ for water is zero.

The difference in strength of the interaction between transported molecules can also explain the different time-transient behaviors in Figure 12. As we discuss in Section 3.2.4, the time-transient behavior of water is well fitted to a linear function of time, although some deviations from linearity occur when the interaction between water molecules and osmolytes is very strong. This linearity implies a constant transport rate of water molecules, which means that this rate does not depend on the concentration of water in Compartment 1 (zeroth-order rate). Physically, this occurs because water molecules tend to aggregate, so the local concentration of water is almost constant regardless of the available space in Compartment 1 (see the inset of Figure 11a). In other words, near the CNT, water molecules are continuously supplied for transport, compensating for the loss of molecules due to transport. In a sense, water transport through a nanochannel driven by osmosis is similar to pulling a string at a constant speed. 

However, as we discussed in our previous work [1], the time-transient behavior of charge-removed water is well fitted to an exponential function of time, not a linear function, because the transport rate is proportional to the concentration (first-order rate). For the transport to occur, charge-removed water molecules in Compartment 1 should approach the CNT, and the probability of going into the CNT is proportional to the number of molecules in Compartment 1. Thus, as more molecules are transferred to Compartment 2, the concentration of molecules in Compartment 1 is reduced, and as a result, the transport rate decreases with time. Particularly, if we assume that the rate is proportional to the concentration of charge-removed water, then we mathematically show that the transient behavior exactly follows an exponential function of time. Therefore, the comparison in Figure 12 indicates that the interaction between transported molecules apparently affects the transport kinetics.

## 4. Summary and Conclusions

In this work, we have studied the transport of water molecules from a nanometer-sized compartment (Compartment 1) to another same-size empty compartment (Compartment 2) through a CNT using MD simulations. If the transported molecules are gas molecules, which weakly interact with each other, then transport would spontaneously occur such that almost half of the gas molecules would be in one compartment and the other half would be in the other compartment. However, for water molecules, transport to Compartment 2 does not occur without any external force. This is because the chemical potential of water in Compartment 2 is higher than that in Compartment 1 and the CNT. 

To induce water transport, we used an osmotic force as an external force by introducing osmolytes in Compartment 2. However, the attractive interactions between Compartment 1 and water molecules can terminate the water transport before all water molecules in Compartment 1 are transported such that some of the water molecules remain next to the walls of Compartment 1. Thus, to transport more water molecules, we weakened the compartment-water interaction by reducing the LJ interaction parameter. With osmolytes in Compartment 2 and weak compartment-water interactions, we were able to observe complete or nearly complete transfer of water molecules to Compartment 2 from extensive MD simulation studies. 

To systematically study the effect of osmolytes, we considered various osmotic environments characterized by the number of osmolytes (*N_O_*) and the strength of the interaction between osmolytes and water molecules (*IS_WO_*). The extensive case studies indicate that *N_O_* and *IS_WO_* appear to have effective threshold values for water transport to occur within a certain time frame in that when *N_O_* and *IS_WO_* are sufficiently large, water transport is always observed in the simulations. However, the occurrence of water transport is intrinsically stochastic, and thus, in practice, the chance to observe water transport depends on the length of the simulation time and the number of simulations. For example, for a given system, if one performs more simulations for a longer simulation time, then observation of water transport is more probable. 

As a result of water transport, the system transitions from the initial state to a stable state. We found that depending on *N_O_* and *IS_WO_*, mainly three kinetically stable states (States I, II, and III) are observed. In the presence of osmolytes in Compartment 2, it is more energetically favorable for water molecules to be in Compartment 2 with osmolytes (State II or State III depending on the occupancy of the CNT) than in Compartment 1 (State I). Therefore, the water transport leading to State II or State III is thermodynamically favorable, but the stability of State I observed in our simulations indicates the existence of a kinetic barrier for the transition to State II or State III, which is the physical origin of the stochastic behavior in the occurrence of water transport. 

We also investigate the transition process from the initial state to State II or State III, in which complete or nearly complete transfer of water molecules to Compartment 2 occurs. As expected, as the number of osmolytes increases, the transition time decreases while the transport rate increases. Interestingly, the interaction strength of osmolytes *IS_WO_* appears to be crucial in the initiation of the process, but once the transition process is initiated, the number of osmolytes *N_O_* contributes more to determining the transport rate. Additionally, as *IS_WO_* and *N_O_* increase, the transport rate seems to saturate. 

One of the most interesting findings from this work is that the kinetics of water transport is zeroth-order, while the kinetics of charge-removed water transport is first-order. Here, water and charge-removed water (water without electric charge) represent strongly and weakly interacting molecules, respectively. In other words, the strength of the interaction between the transported molecules can significantly affect the transient behavior of the transport. Physically, the zeroth-order kinetics in water transport means a constant flow of water, which is possible due to the strong attractions between water molecules; one molecule is followed by another, and in this sense, water transport through a nanochannel by osmosis is similar to pulling a string at a constant speed.

In this work, we used a minimal model system that has only the components necessary for producing water transport through a nanochannel by osmosis. Therefore, based on this basic model, we can extend the study to various related topics by modifying the model. For example, in the basic model, we used nonpolar osmolytes, and therefore the interactions between water molecules and osmolytes were simply described by the LJ interactions. However, in reality, osmolytes could be polar or charged molecules. Therefore, in future studies, water transport driven by charged osmolytes could be an interesting subject. Another interesting subject would be the water transport and equilibrium states for the system containing osmolytes in Compartment 1 as well as in Compartment 2. 

## Figures and Tables

**Table 1:** Kinetically stable states observed from the simulations in Figure 4.

	*NO*	10	25	50	75	100	125
*IS*_*WO*_							
0.1		State I	State I	State I	State I	State I	State I
1		State I	State I	State I	State I	State I	State I
5		State I	State I	State I	State I	State I	State I
10		State I	State I	State I	State II	State II	State II
20		metastable	State II	State II	State II	State II	State III
50		State II	State II	State II	State II	State III	State III
